# Chromatin remodeller Chd7 is developmentally regulated in the neural crest by tissue-specific transcription factors

**DOI:** 10.1371/journal.pbio.3002786

**Published:** 2024-10-17

**Authors:** Ruth M. Williams, Guneş Taylor, Irving T. C. Ling, Ivan Candido-Ferreira, Daniel M. Fountain, Sarah Mayes, Perihan Seda Ateş-Kalkan, Julianna O. Haug, Andrew J. Price, Sean A. McKinney, Yavor K. Bozhilovh, Richard C. V. Tyser, Shankar Srinivas, Jim R. Hughes, Tatjana Sauka-Spengler

**Affiliations:** 1 Stowers Institute for Medical Research, Kansas City, Missouri, United States of America; 2 University of Oxford, MRC Weatherall Institute of Molecular Medicine, Radcliffe Department of Medicine, Oxford, United Kingdom; 3 University of Oxford, Department of Paediatric Surgery, Children’s Hospital Oxford, Oxford, United Kingdom; 4 University of Oxford, MRC WIMM Centre for Computational Biology, MRC Weatherall Institute of Molecular Medicine, Oxford, United Kingdom; 5 University of Oxford, MRC Molecular Haematology Unit, MRC Weatherall Institute of Molecular Medicine, Radcliffe Department of Medicine, Oxford, United Kingdom; 6 University of Oxford, Department of Physiology, Anatomy and Genetics, South Parks Road, Oxford, United Kingdom; Institute of Science and Technology Austria, AUSTRIA

## Abstract

Neurocristopathies such as CHARGE syndrome result from aberrant neural crest development. A large proportion of CHARGE cases are attributed to pathogenic variants in the gene encoding CHD7, chromodomain helicase DNA binding protein 7, which remodels chromatin. While the role for CHD7 in neural crest development is well documented, how this factor is specifically up-regulated in neural crest cells is not understood. Here, we use epigenomic profiling of chick and human neural crest to identify a cohort of enhancers regulating *Chd7* expression in neural crest cells and other tissues. We functionally validate upstream transcription factor binding at candidate enhancers, revealing novel epistatic relationships between neural crest master regulators and Chd7, showing tissue-specific regulation of a globally acting chromatin remodeller. Furthermore, we find conserved enhancer features in human embryonic epigenomic data and validate the activity of the human equivalent *CHD7* enhancers in the chick embryo. Our findings embed *Chd7* in the neural crest gene regulatory network and offer potentially clinically relevant elements for interpreting CHARGE syndrome cases without causative allocation.

## Abstract 2

CHARGE syndrome results from aberrant neural crest development, affecting eye, heart, and facial structures, and has been attributed to pathogenic variants of the chromatin remodeler CHD7. This study characterizes the enhancers and tissue specific transcription factors that regulate Chd7 expression in the developing chick and human neural crest.

## Introduction

The neural crest is a transient and migratory embryonic progenitor population that contributes to a remarkable range of neural and mesenchymal tissues in the vertebrate body. Neural-neural crest derivatives include facial ganglia, and both neurons and glia of the peripheral and enteric nervous systems. Mesenchymal neural crest derivatives include craniofacial cartilage and bone, as well as smooth muscle of facial blood vessels, striated muscle forming the cardiac outflow tract and septal, and the majority of the body’s pigment cells. Consequently, errors in neural crest patterning, migration, and differentiation result in a wide range of congenital birth anomalies collectively termed neurocristopathies. Neurocristopathies account for almost one third of all birth defects [1]. These include CHARGE syndrome, which affects the eye, heart, and facial structures [2,3]; Hirschsprung’s disease, characterised by the loss of neural crest-derived enteric ganglia [4,5]; Waardenburg syndrome, characterised by deafness, pigmentation, and craniofacial defects [6]; and Treacher Collins syndrome presenting with craniofacial defects [7,8]. Neurocristopathies can be caused by pathogenic variants of master neural crest regulators, for example, SOX10 in Hirschsprung’s disease and Waardenburg syndrome [9–13] or PAX3 in Waardenburg syndrome [14–16]. However, mutations in genes encoding general cellular machinery can also result in neurocristopathies as demonstrated in Treacher Collins syndrome caused by pathogenic variants of RNA polymerase I [8] and CHARGE syndrome where heterozygous mutations in the chromatin remodeller *CHD7* (chromodomain helicase DNA binding protein 7) are detected [17].

CHARGE syndrome (OMIM 214800) individuals present with ocular Coloboma, Heart malformations, choanal Atresia, Retardation of growth, Genital hypoplasia, and Ear abnormalities. Over 500 different pathogenic variants of CHD7 have been described, accounting for >90% of CHARGE syndrome cases [18,19]. However, in routine clinical screening, mutations in *Chd7* are only detected in 32% to 41% of suspected CHARGE cases [18]. Pathogenic variants have been reported throughout the *CHD7* gene body, indicating premature termination of the protein is significantly detrimental to CHD7 function [18–22]. CHD7 influences gene regulation [18,23] by catalysing nucleosome repositioning in an ATP-dependent manner [24]. In keeping with this regulatory role, CHD7 associates with distal regulatory sites carrying H3K4me1 chromatin modifications characteristic of poised enhancers in mouse embryonic stem cells [25].

Previous studies have shown that CHD7 function is essential for proper neural crest development and migration. In cell models of human neural crest cells, CHD7 occupies distal regulatory elements for neural crest transcription factors SOX9 and TWIST1 [26]. In mice, *Chd7* heterozygotes present with CHARGE syndrome-like features [27–29] and trunk neural crest cells require *Chd7* to maintain their multipotency [30,31]. In Xenopus, *Chd7* mutant embryos have reduced *Sox9*, *Twist1*, and *Snai2* expression and display CHARGE syndrome-like features [26]. Recent work in the chick neural crest, employing weighted gene co-expression network analysis (WGCNA) analysis [32], showed that *Chd7* expression strongly correlated with expression of neural crest regulators (*Sox5*, *Sox9*, *Zeb2*, and *NeuroD4*), other chromatin remodellers (*Kdm1B*, *Kdm2A*, *Kdm3B*, *Kdm7A*) and Semaphorins (*Sema3A*, *Sema 3E*, *Sema4D*, *Sema6D*) which have previously been shown to be regulated by *Chd7* in mice [33]. Collectively, these findings provide strong evidence for positioning *Chd7* within the neural crest gene regulatory network. However, the regulatory mechanisms governing *Chd7* up-regulation in the neural crest have not been investigated. Since CHD7 is a component of the general cellular epigenetic machinery and is broadly expressed in a multitude of embryonic tissues, it could be assumed that its expression is governed by basal, proximal enhancers; however, the clinical features of CHARGE syndrome clearly suggest CHD7 has tissue and developmental stage-specific roles [3].

Here, we identify multiple novel enhancers driving *Chd7* expression in developing chick embryos. We validate enhancer activity in vivo and functionally determine key upstream transcription factors mediating *Chd7* enhancer activity in the neural crest. Using human embryonic chromatin accessibility data, we find that neural crest-specific *CHD7* enhancers are highly conserved. Furthermore, we demonstrate that human enhancers are active in developing chick embryos, suggesting that the tissue-specific regulatory mechanisms enhancing *Chd7* in the neural crest are conserved between chicken and human. Our findings provide a potential mechanism to explain the aetiology of CHARGE syndrome where the *Chd7* gene itself is unperturbed. Finally, we demonstrate the upstream regulation of a chromatin-remodeller by tissue-specific transcription factors as an important mechanism to ensure enhanced chromatin remodelling activity in the developing neural crest.

## Results

### Chd7 expression during early chick development

**Fig 1:**
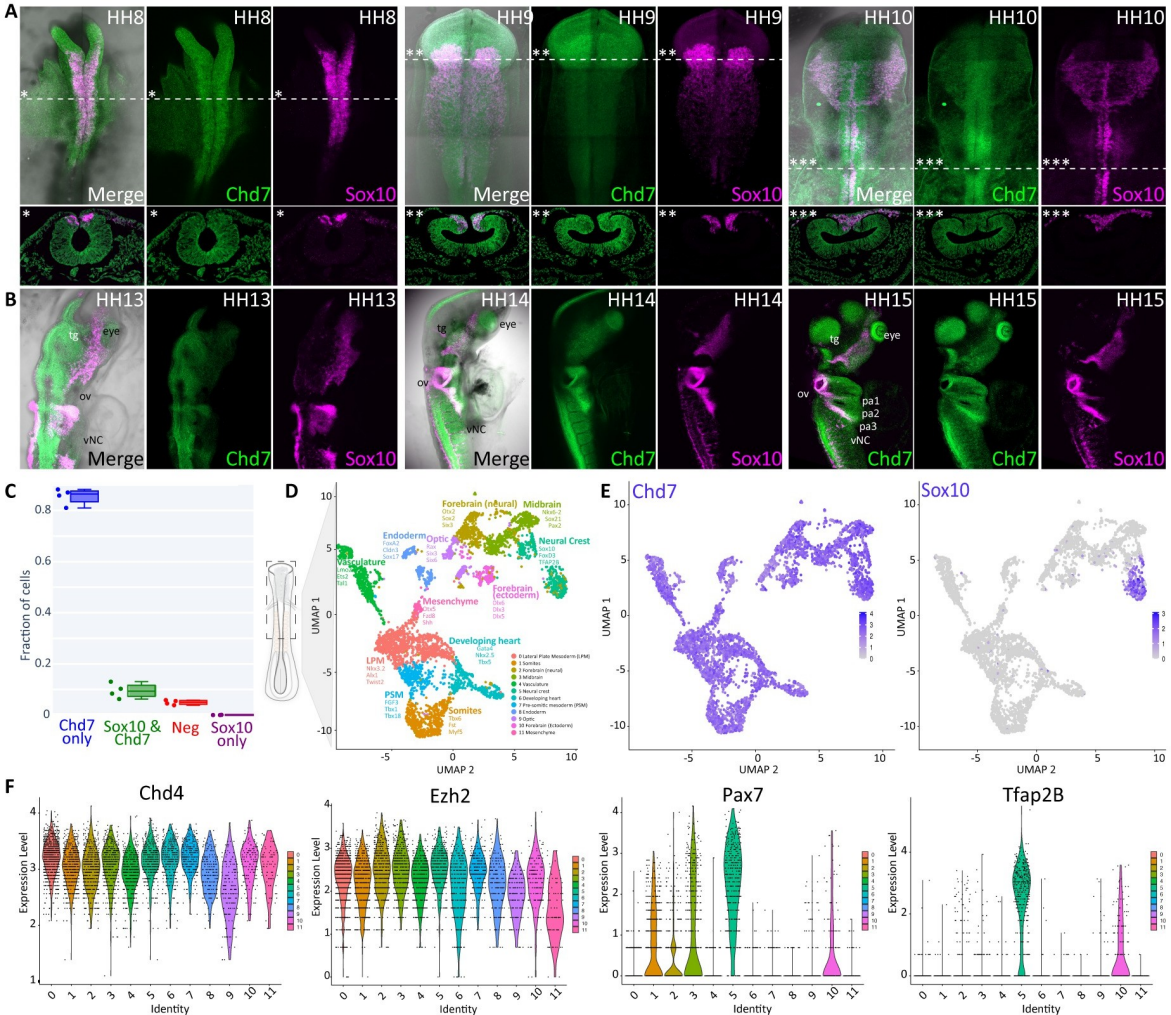
Chd7 expression during early chick development. (A, B) In vivo *Chd7* expression (green) determined using HCR. *Sox10* (magenta) is used as a neural crest marker. (A) *Chd7* is expressed in neural crest cells and throughout the neural tube, as well as the surrounding ectoderm and underlying mesoderm at HH8-10. (B) *Chd7* is more broadly expressed at HH13-HH15 including head and hindbrain structures. From HH13 through HH15 *Chd7* expression is detected in the otic vesicle (ov), trigeminal ganglia (tg) eye, and vagal neural crest (vNC). At HH15 *Chd7* is also expressed in the pharyngeal arches 1–4 (pa1-4) and dorsal root ganglion (drg). (C) Quantification of HCR signal from *Chd7* and *Sox10* transcripts in stage HH10 chick embryos (*n* = 4). (D) UMAP representation of 5,669 single cells resolved into 12 clusters of based on shared transcriptional identities. (E) Feature plots of *Chd7* and *Sox10* expression across scRNA-seq clusters. (F) Violin plots of selected chromatin remodellers and transcription factors expressed across scRNA-seq clusters. HCR, hybridisation chain reaction.

*Chd7* was previously shown to be enriched in bulk RNA-seq data from chick cranial neural crest cells [32] where it was co-expressed with other neural crest genes and clustered with pre-migratory neural crest markers https://livedataoxford.shinyapps.io/Chick_NC_GRN-TSS-Lab/. Here, we used fluorescent in situ hybridisation (hybridisation chain reaction, HCR) [34] to resolve spatiotemporal *Chd7* expression in developing chicken embryos. *Chd7* was first detected at HH8 [35] within the cranial neural tube and pre-migratory neural crest cells as indicated by colocalisation with the neural crest marker *Sox10* and at lower levels in the surrounding neuroectoderm (Fig 1A). *Chd7* transcripts continued to overlap with *Sox10* in delaminating and migrating neural crest cells at HH9/10 and were also detected in the neuroectoderm, forebrain, and neural tube at the vagal level from HH9 through HH15 (Fig 1A and 1B). At later stages (HH13—HH15) *Chd7* was more broadly expressed, with transcripts distributed across the head regions (midbrain and hindbrain) including the trigeminal ganglia, developing face mesenchyme, eye, and otic vesicle as well as some vagal neural crest cells. In addition, at HH15, pharyngeal arches and dorsal root ganglion cells were also *Chd7* positive (Fig 1B). Quantification of the HCR signal from *Chd7* transcripts revealed approximately 85% of DAPI positive cells expressed *Chd7*, compared to 10% of cells that were *Chd7* and *Sox10* double positive (Fig 1C).

To gauge the dynamics of *Chd7* expression in individual cells in the entire embryo, we performed single-cell RNA-seq using the 10X Genomics 3’ scRNA-seq platform. We dissected the anterior half of 10 HH10 chick embryos (Fig 1D) and obtained 5,669 single-cell transcriptomes, resolved into 12 clusters (Fig 1D). *Chd7* expression was detected in all clusters (Fig 1E), comparable with other chromatin remodellers (Figs 1F and S1A) and in contrast to cluster-specific expression of bona fide transcription factors (Figs 1F and S1B). Crucially, this suggests the inherent differences in the regulatory mechanisms controlling the dynamic expression of tissue-specific transcription factors and more broadly expressed chromatin regulators.

### Epigenomic annotation of the *Chd7* locus identifies numerous enhancer elements

**Fig 2:**
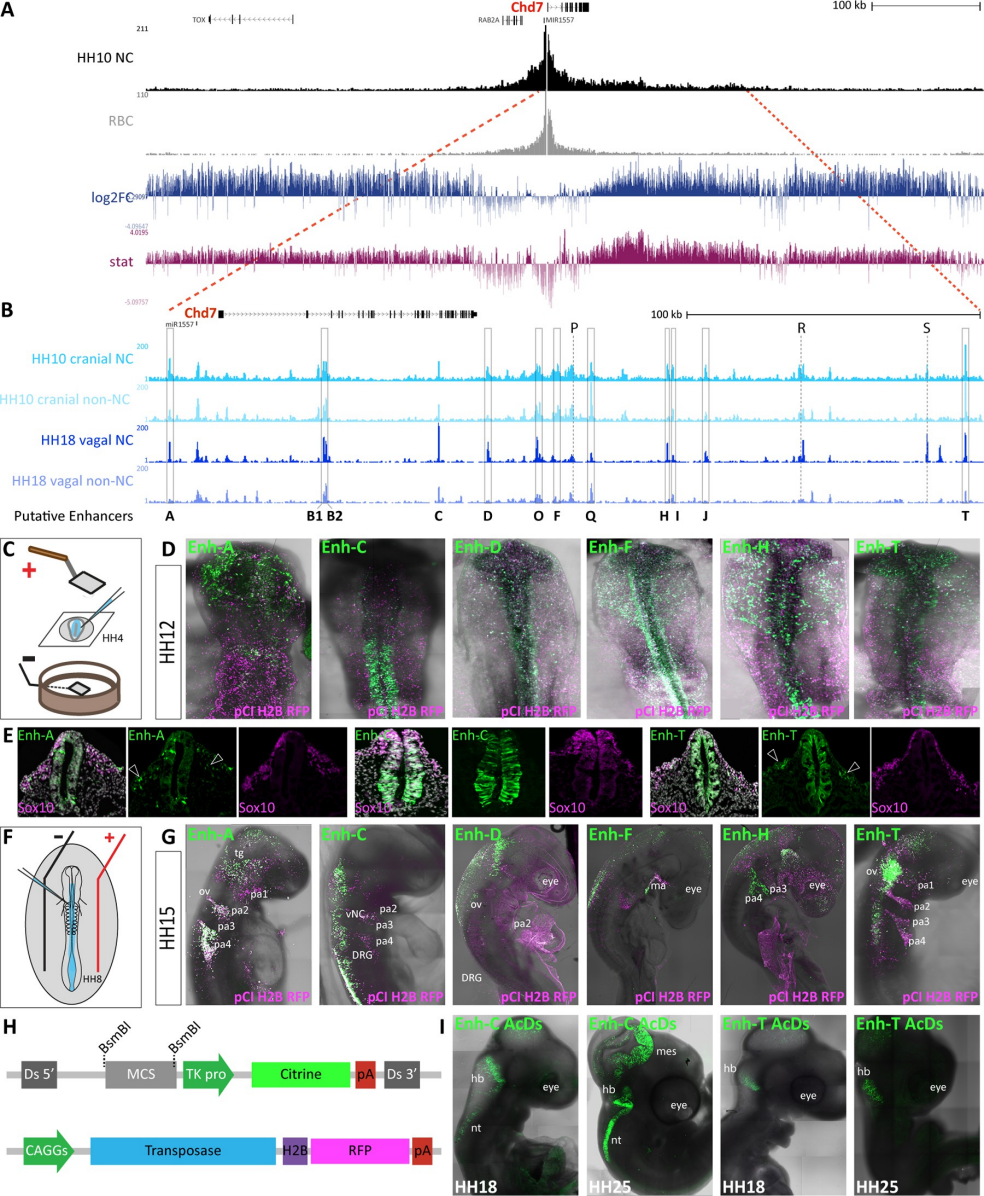
*Chd7* enhancer prediction from neural crest Capture-C and ATAC data. (A, B) UCSC genome browser view of the chick *Chd7* locus in galGal5. (A) Capture-C tracks from cranial neural crest at HH10 and control RBC, showing the *Chd7* TAD. Differential interactions were determined using DESeq2, hypothesis tested with Wald test and corrected for multiple testing using the Benjamin–Hochberg method. Wald statistics track (stat, in pink) represents ratio of LogFoldChange values and their standard errors. (B) ATAC-seq data from HH10 cranial [32] and HH18 vagal [37] neural crest cells and non-neural crest control cells collected by FACS using *FoxD3* enhancer NC1 (cranial) and *FoxD3/Ednrb* enhancers NC2/E1, respectively. Putative enhancers within the *Chd7* TAD are indicated by grey boxes. (C) Schematic representation of ex ovo chicken embryo electroporation technique used to deliver enhancer reporter constructs. (D) Fluorescent reporter activity (green) recorded from indicated enhancers at HH12 following ex ovo electroporation at HH4 with a ubiquitous electroporation control pCI-H2B-RFP (magenta). (E) Transverse sections of embryos at approximately HH12 showing enhancer activity in green and *Sox10* expression in magenta, detected by HCR. Migrating neural crest cells are indicated with arrows. (F) Schematic representation of in ovo chicken embryo electroporation. (G) Enhancer activity (green) recorded from indicated enhancers at HH15 following in ovo electroporation into the neural tube at HH9 with a ubiquitous electroporation control pCI-H2B-RFP (magenta). pa; pharyngeal arch, tg; trigeminal ganglia, ov; otic vesicle, ma, mandibula arch. (H) Schematics of AcDs constructs used to integrate the enhancer reporter cassette into the genome to observe sustained enhancer activity in vivo. (I) Enhancer activity of indicated enhancers at later stages following in ovo electroporation of AcDs enhancer reporter plasmids, nt; neural tube, mes; mesencephalon, hb; hindbrain. HCR, hybridisation chain reaction; RBC, red blood cell; TAD, topologically associating domain.

*Chd7* was broadly expressed during early chick development, consistent with its function as a chromatin remodeller; however, *Chd7* transcripts were notably enriched in the developing neural crest in keeping with its known role in CHARGE syndrome. We next queried whether such a pervasive factor may be regulated in a tissue-specific fashion in the neural crest. To this end, we explored neural crest-specific epigenomic features depicting putative enhancers interacting with the *Chd7* promoter. We first determined the *Chd7* topologically associating domain (TAD) using Next-Generation Capture-C [36], a high-resolution targeted 3C approach adapted for low cell numbers. Using dissected dorsal neural tube tissue from HH8-10 chick embryos and differentiated red blood cells (RBCs) from 10-day-old chick embryos as controls, we resolved a broad neural crest-specific TAD of approximately 1.1 Mb, spanning approximately 85 Kb upstream and approximately 1 Mb downstream from the *Chd7* promoter, encompassing the entire gene body (Fig 2A).

We next surveyed the chromatin accessibility landscape within the *Chd7* TAD using ATAC-seq data obtained from chick cranial [32] and vagal [37] neural crest and non-neural crest control cells at HH10/HH18, respectively (Fig 2B). We selected putative *Chd7* enhancers based on chromatin accessibility in neural crest cells. In total 14 putative enhancers were selected (A, B1/B2, C, D, F, H, I, J, O, P, Q, R, S, T) and screened for in vivo enhancer activity, using a fluorescent enhancer reporter assay [32,38]. In order to determine the full temporal range of enhancer activity, we performed both ex ovo electroporation at HH4, where the whole embryo is targeted, but development time post-electroporation is limited (Fig 2C) and in ovo electroporation at HH8 which allows for analysis further into development but only targets the neural tube and neural crest (Fig 2F) [39]. These assays revealed 11 active enhancers (Figs 2D–2G and S2). Enhancers (enh) A, D, F, H, and T were all active in delaminating and migrating cranial neural crest cells at HH10-12 (Figs 2D, 2E and S2), as determined by the colocalisation of their activities with the expression of the neural crest marker *Sox10* (Figs 2E and S3). Activities of enhancers F, H, and T were also seen in the neural tube (Figs 2D, 2E, and S2), whereas enh-D was broadly active across the neuroectoderm, consistent with the accessibility profiles of these elements in non-neural crest cells (Fig 2B). At HH15 enhancers A, D, F, H, and T were dynamically active in the head mesenchyme (Fig 2G). At this stage, enhancers A, H, and T were also active in the trigeminal ganglia and migrating cardiac neural crest cells as they migrated into the pharyngeal arches (Fig 2G). We did not observe any enhancer activity in the developing heart itself, consistent with the lack of cardiac *Chd7* expression at the stages studied. It is likely that heart specific *cis*-regulation initiates at later stages activating *Chd7* in this tissue [40]. Enh-C was also active in the pharyngeal arches but was predominantly active in the neural tube and premigratory neural crest cells extending posteriorly from the hindbrain at HH10-15 (Figs 2D, 2E and S2). At HH15 enh-C activity was also seen in the dorsal root ganglia (DRG) (Fig 2G).

We also tested 2 large ATAC peaks (B1 and B2) located within the second intron of the *Chd7* gene. We found that enh-B1 exhibited mosaic activity in pre-migratory neural crest cells at HH8 and in neural tissue at HH12 (S2 Fig). Enh-B2 was predominantly active in the otic placode region at HH12 and later in the hindbrain and the neural tube at the vagal level (HH25) (S2 Fig). Enh-Q (−127 kb) was also active in the neural tube at vagal and cervical levels as well as in a small number of cells in the otic placode region at HH10-12. Enh-S activity was restricted to the otic placode (S2 Fig).

Enh-O activity was confined to the cranial neural tube at HH8-10 but was active across the cranial region, including in the migrating neural crest cells at HH12. Later, (HH18) enh-O activity was also detected in the vagal neural tube (S2 Fig), consistent with accessibility in the vagal neural crest as well as non-neural crest at this stage (Fig 2B). Enh-I, also accessible in both neural crest and non-neural crest cells and was active in a broad embryonic territory at HH10-12 (S2 Fig). Enh-J was also broadly active across the head at HH12 but confined to the face mesenchyme and trunk at HH15 (S2 Fig). Other putative elements tested (enh-P and enh-R) (Fig 2B) showed no enhancer activity.

Since our original enhancer reporter system is episomal, reporter constructs are subject to degradation and dilution as development proceeds. Thus, interpreting enhancer activity at later time points is problematic. To circumvent this, we adapted the AcDs maize integration system [41] to the chicken model. In this system, the enhancer reporter cassette is flanked by Ds recombination sites and co-electroporated with a plasmid expressing Ac transposase ubiquitously, resulting in the random integration of the cassette into the genome (Fig 2H), and subsequent stabilisation of enhancer activity in cells expressing cognate transcription factors. Since integration is random, it is possible that some enhancer activity is not recorded or appears weaker due to chromatin position effects; however, coupled with our episomal assays this system provides a reliable read-out for enhancer activity at later time points. We selected enh-C and enh-T for this approach because these regions were highly accessible in our HH18 data and therefore likely to be active at later stages. We followed enhancer activity until 4 days post in ovo electroporation. Enh-C activity was detected in the mesencephalon, hindbrain, and neural tube, while enh-T was active in a discreet region of the hindbrain (Fig 2I).

By conducting a thorough survey of *Chd7* epigenomic landscape in the chick embryo, we revealed a large cohort of enhancers putatively controlling *Chd7* expression. Interestingly, we identified several enhancers specifically active in the neural crest and others displaying broader activity in other *Chd7* positive tissues, thus demonstrating differential tissue-specific regulation.

### Conservation of Chd7 enhancer activity in human

**Fig 3:**
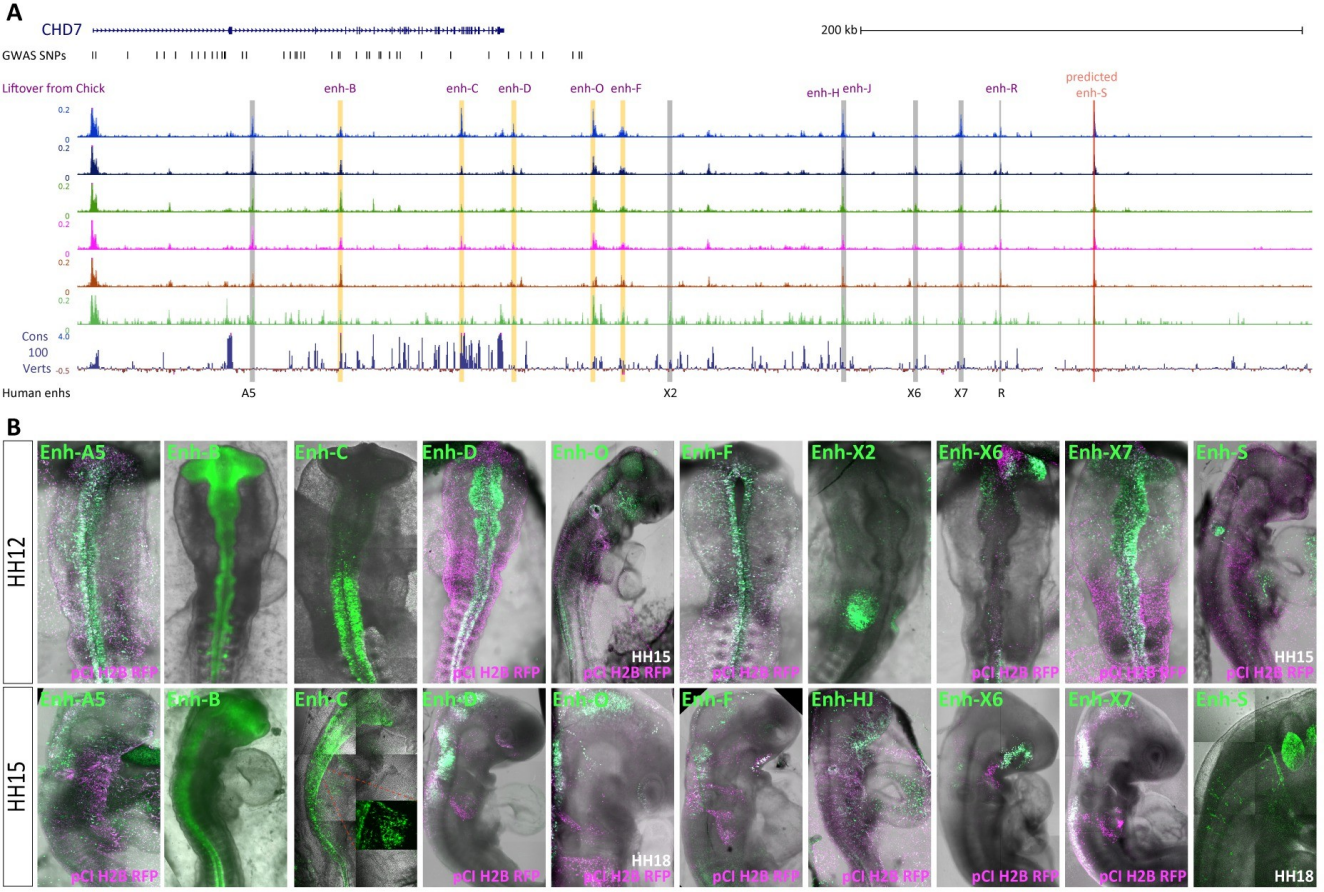
Epigenomic landscape of human CHD7 locus. (A) CHD7 locus in human genome (hg38, chr8:60,665,230–61,241,522), showing ATAC profiles of 6 clusters from Multiome data generated from human embryonic cranial samples (see Methods). Chick enhancers lifted over from galGal5 are shown in orange boxes; novel human enhancers are shown in grey boxes. GWAS SNPs from the NHGRI-EBI GWAS Catalog is also indicated. (B) Human enhancer activity (green) in vivo at HH12 following ex ovo electroporation and HH15 following in ovo electroporation. Ubiquitous electroporation control pCI-H2B-RFP is shown in magenta. GWAS, genome-wide association studies.

While a large proportion of CHARGE syndrome cases are directly linked to pathogenic variants of *CHD7* gene itself, there remain a significant number of cases without causal annotation. We postulated that some instances of CHARGE syndrome may be indirectly linked to *CHD7*, via perturbation of upstream regulatory mechanisms controlling *CHD7* expression. To this end, we sought to identify regulatory elements controlling the expression of CHD7 in human. We used 10X Multiome data (simultaneous gene expression and chromatin accessibility single-cell profiling) generated for another study from cranial tissue samples at CS16/18/19 (5 to 6.5 weeks), which is analogues to chick HH25-HH28. Following quality control (see Methods), we resolved 33 clusters from 14,290 nuclei (S4A Fig). *CHD7* was broadly expressed across all clusters (S4B Fig), comparable to other chromatin remodellers and in contrast to tissue-specific TFs (S4B and S4C Fig). We mapped the homologues of chick *Chd7* enhancers to the human ATAC data using the LiftOver function from UCSC genome browser (Fig 3A). We identified 5 accessible elements in the human data corresponding to chick *Chd7* enhancers, B, C, D, O, and F (Fig 3A) and tested these human homologous sequences using the chick enhancer reporter assay (Fig 3B). Human enhancers C and F activity strictly recapitulated that of their chicken counterparts. Enh-B and enh-D were more restricted to neural tissue compared to the chick equivalents, with enh-D activity detected as particularly strong in the hindbrain and mesencephalon. Human enh-O was also active in the hindbrain and mesencephalon (HH15-HH18) as well as across the face mesenchyme, otic placode (at HH15) (Fig 3B). Enhancers -B and -C were well conserved within vertebrates (PhastCons score [42] 0.91 and 0.73, respectively, S2 File), and these elements were active in the trunk neural tube. Thus, the conservation of these elements is consistent with the notion that trunk neural crest gene regulatory elements are more conserved than those in the cranial neural crest [43]. From these human ATAC-seq data, we also cloned and tested more than 20 putative regulatory elements and identified 5 novel tissue-specific human *CHD7* enhancers. Enh-A5 and enh-X7 were predominantly active in the neural tube but also present in the surrounding ectoderm including neural crest cells at HH12 (Fig 3B). At HH15, enh-X7 was strong in the hindbrain and neural tube (Fig 3B). Human enh-X2 and enh-S were predominantly active in the otic placode. These elements showed increased accessibility in cluster-6 particularly enh-X2 which was exclusively open here. Enh-X2 was well conserved (PhastCons score 0.53), but since our chick data was generated from neural crest cells and this element appears to be otic specific, it was not detected in the chick data. Human enh-X6 also presented a restricted accessibility profile in clusters 10 and 11 and activity was restricted to the optic region (Fig 3B).

We also explored previously published epigenomic data obtained from in vitro derived human cranial neural crest cells [44]. Specifically, we examined ChIP-seq data for histone modifications associated with active enhancers (H3K4me1, H3K27ac), promoters (H3K4me3), and repressed chromatin (H3K27me3), as well as ATAC-seq data (S4D Fig). All tested enhancers were marked by H3K4me1, (S4D Fig) with the exception of enh-C; however, this data was generated from cranial neural crest cells and enh-C was primarily active in the trunk (Fig 3B). Most enhancers showed some accessibility in the in vitro ATAC data, (A5, D and F being the exceptions). Enh-B and enh-O were marked by H3K27ac, enh-O was also marked by H3K4me3, which was also present at enh-S (S4D Fig). Enh-S also had H3K27me3 enrichment, since enh-S activity is restricted to the otic placode, it is plausible that this region is repressed/compacted in other cell types. The same can be said for enh-F (S4D Fig). Collectively, our observations from these data corroborate our in vivo enhancer reporter assay results.

To explore the potential association between CHD7 enhancers and CHARGE syndrome, we surveyed data from the NHGRI-EBI Catalogue of human genome-wide association studies (GWAS) (https://www.ebi.ac.uk/gwas/genes/CHD7) in search of SNPs within CHD7 enhancers. We found most SNPs were located within the gene body of CHD7 (Fig 3A), two of which overlapped with enh-B. Others were in close proximity to enh-C and enh-D. This suggested that CHD7 enhancers lie within genomic regions already shown to be susceptible to alterations implicated in CHARGE syndrome. It has recently been shown that changes at the level of individual SNPs within a single TF motif cause minimal to moderate alterations in binding affinity but can have a significant influence on enhancer activity [45]. Thus, more extensive analysis of SNPs in CHARGE patients may yet link enhancer activity to putative pathogenic variants.

By comparing human and chick *CHD7* loci, we demonstrate conserved *cis*-regulation of this gene across amniotes. Our findings illustrate the utility of in vitro derived data, while highlighting important differences observed within the in vivo embryonic context. Furthermore, our findings provide an alternative mechanism by which *CHD7* expression can be altered in CHARGE individuals, where pathogenic variations are not detected in the gene itself, but in its regulatory elements.

### Chd7 enhancers are differentially regulated by neural crest transcription factors

**Fig 4:**
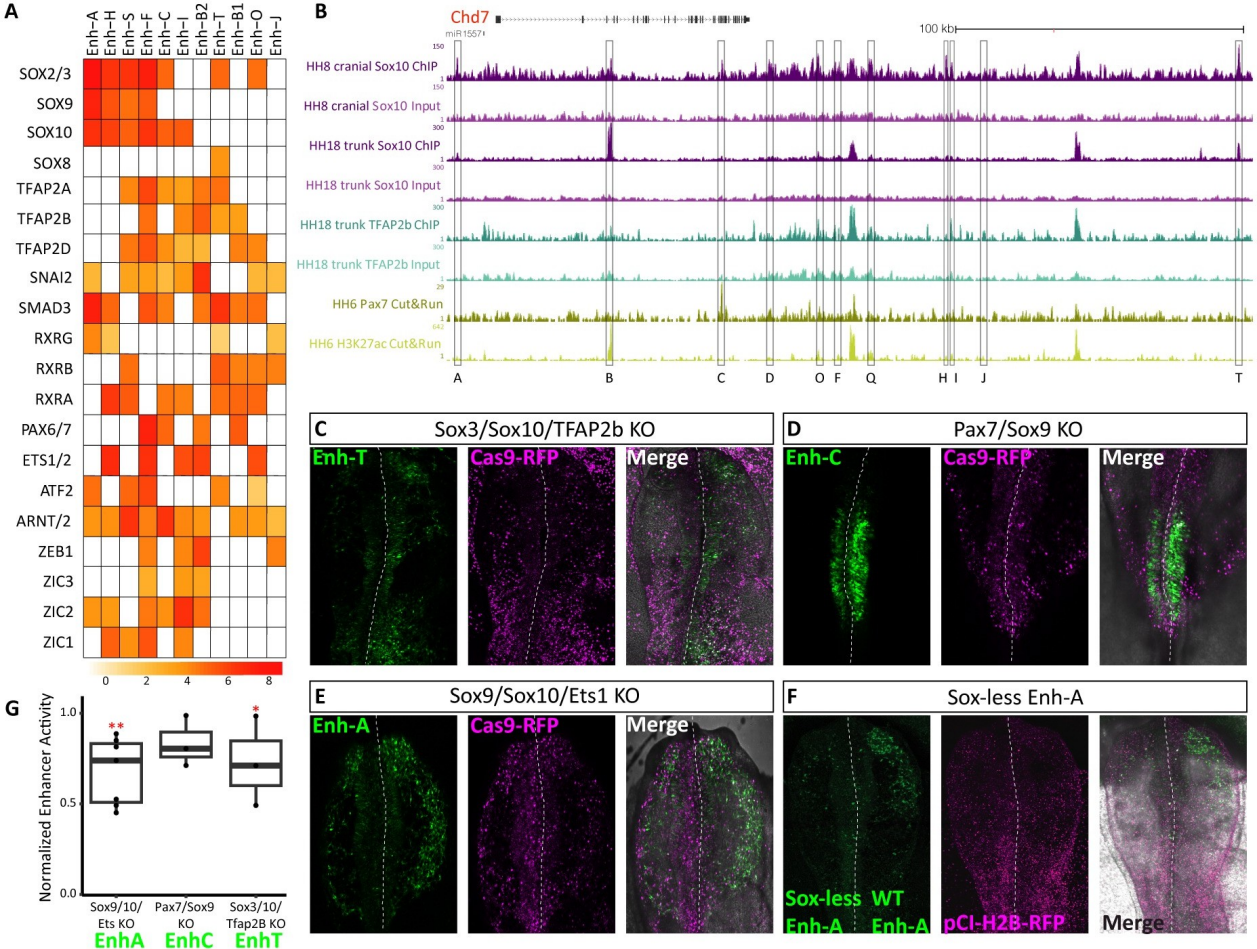
Upstream transcription factors controlling Chd7 enhancer activity. (A) Heatmap showing predicted TF binding motifs within chick *Chd7* enhancers. (B) Tracks from UCSC genome browser showing biotin-ChIP data for Sox10 (purple), Tfap2B (teal), Pax7 (yellow), and H3K27ac. (C–E) Chicken embryos showing indicated enhancer activity (GFP) and Cas9 (RFP) following bilateral electroporation of Cas9 and guide RNAs targeting selected TFs on the left (experimental) and scrambled guide RNA with Cas9 on the right (control). (F) Chicken embryo bilaterally electroporated with a construct containing enh-A where all the Sox sites have been mutated on the left and wild-type enh-A construct on the right, both driving GFP expression. pCI-H2B-RFP was co-electroporated on both sides as a control. (G) Loss of enhancer activity (GFP intensity) following TF knockout experiments in (C–E) was quantified left versus right, relative to control RFP intensity, two-tailed paired *T* test, enh-A; *p* = 0.023**, *n* = 7 embryos, enh-C; *p* = 0.114, ns *n* = 3, enh-T; *p* = 0.047*, *n* = 4. TF, transcription factor.

Enhancer activity is determined by the combinatorial binding of transcription factors (TFs) to their cognate motifs within the enhancer sequence. Indeed, such interactions are the foundation for cell type-specific regulation of gene expression. In order to explore putative TF binding within the chick *Chd7* enhancers, we firstly scanned human TFs binding motifs, allowing us to identify motif instances within each enhancer sequence (S5 Fig). Next, we calculated the enrichment of binding sites within each enhancer using the log odds ratio score (Fig 4A). The 2 approaches yielded broadly similar results. Across all chick *Chd7* enhancers, we found an enrichment of neural crest associated TF’s, such as Tfap2, SoxE (Sox8/9/10) and SoxB1 (Sox2/3) factors as well as a significant presence of Zic family members (Fig 4A). Many enhancers also contained motifs for Arnt/Arnt2 and ATF2 factors, which we previously described in our survey of core upstream TF’s driving neural crest specification [32]. Given the broad spatial and temporal range of *Chd7* enhancers, it is likely that different TF combinations drive enhancer activity at different time points and in different embryonic locations. In particular, we noted that the neural crest-specific enhancers enh-A and enh-T were differentially enriched for SoxE and Tfap2 factors, respectively (Figs 4A and S5), suggesting differential mechanisms for *Chd7* regulation within the same cell population (cranial neural crest). Enh-H shared a similar signature with enh-A, whereby both enhancers lack Tfap2 sites but are enriched for Sox9/10 and Sox2/3 sites. Enh-A, enh-F and enh-C were enriched for the motifs of transcriptional repressor Snai2 that likely prevents enh-A activity in the hindbrain region, where Snai2 is highly expressed. Such repressive activity is likely circumvented in enh-C and enh-F elements by Pax6/7, whose motifs are notably absent from enh-A (Fig 4A). Furthermore, Pax6/7 expression overlaps with both enh-F and enh-C activities, found in the neural tube from the hindbrain region and more posteriorly at HH12 (Fig 2D). At HH15 enh-F is also active in the developing eye consistent with *Pax6* co-expression in this region (Fig 2D). Conversely, enh-C activity persists in the trunk neural tube (Fig 2D) consistent with *Pax7* co-expression in this territory. Thus, the Pax binding motif likely accounts for differential spatiotemporal regulation of these enhancers by cognate factors. Enh-F and enh-S were enriched for both SoxE/B and Tfap2 motifs; however, enh-S displayed no enhancer activity in the chick embryo, potentially due to RXR mediated chromatin compaction, since these motifs were present in enh-S, but not in enh-F. While Zic factors are broadly expressed in the neural, placodal, and neural crest territories, their binding motifs were largely absent from neural crest enhancers (enh-A, T, C). Only enh-F, which was active in early cranial neural crest cells as well as some ectoderm cells in the hindbrain region, contained binding sites for all Zic factors (Zic1, 2, 3), suggesting this family regulates non-neural crest enhancer activity, consistent with expression patterns. Motif analysis also implicates Ets1 in *Chd7* regulation, where Ets1 sites were detected in enh-H and enh-F (Fig 4A).

Given previously established relationships between *Chd7* and *Sox10* [46,47], we set out to explore whether Sox10 could also be acting upstream of *Chd7*. Furthermore, since binding sites for specific factors within TF families can be difficult to discern, we sought to validate our TF binding predictions using direct binding assays. First, we profiled Sox10 binding genome-wide using Biotin-Streptavidin based Chromatin Immuno-Precipitation (Biotin-ChIP) [48]. For this purpose, we generated constructs to enable Avi-tagging of transcription factors at the N- or C-terminal. Furthermore, we incorporated an enhancer reporter cassette into the construct to allow for the Avi-tagged-TF to be expressed in a tissue-specific manner under the control of its cognate enhancer, thus mimicking not only spatiotemporal patterns, but also expression levels of the endogenous gene (S5B Fig) (Addgene #110204, #110205, see Methods). In the case of Avi-tagged Sox10, we used the *Sox10* enh-99 (S5C Fig) [32] to drive C-terminally tagged Sox10. Following co-electroporation with ubiquitously expressed biotin ligase, BirA, (Addgene #127781) [37] into the epiblast of stage HH4 chicken embryos the Avi-tagged-TF gets biotinylated in vivo, facilitating highly stringent isolation of the factor with its interacting DNA moieties using streptavidin beads [48]. Hence, this allowed us to isolate specific TF of interest at different stages and examine TF-bound regions genome-wide. Most active *Chd7* enhancers showed some binding by Sox10, compared to the ChIP input sample (Fig 4B), indicating Sox10 is a key player in *Chd7* regulation. Neural crest-specific enhancers, enh-A and enh-T showed particularly high Sox10 occupancy, consistent with our motif analysis (Figs 4A and S5). Enh-H and enh-F were also significantly enriched in Sox10-bound regions, suggesting Sox10 likely contributes to their neural crest-specific activity. Other enhancers whose activity was not restricted to the neural crest including enh-B, enh-C, enh-D, and enh-O were also bound by Sox10 suggesting Sox10 drives their activity in neural crest cells, whereas other factors control their activity in non-neural crest territories.

Tfap2B is a known neural crest specification factor. Given the observed enrichment of Tfap2B motifs across Chd7 enhancers (Fig 4A), we examined direct Tfap2B binding at those elements using Tfap2B biotin-ChIP-seq data generated from cranial and vagal neural crest cells of HH18 embryos [37] (Fig 4B). These data confirmed that Tfap2B indeed binds to enh-T, enh-F, enh-B as well as enh-Q and enh-I as predicted by our motif analysis (Figs 4A and S5A). Interestingly, we did not detect any Tfap2B binding at enh-A, again suggesting differential control of some neural crest-specific *Chd7* enhancers by Sox10 and Tfap2B, most likely is specific neural crest subpopulations and possible future derivatives (Fig 4A). Next, using the Cut&Run (Cleavage Under Targets and Release Using Nuclease) assay [49,50], we also profiled Pax7 binding in dorsal neural tube tissue extracted at HH8-9 (Fig 4B). Here, we confirmed binding of Pax7 at enh-C, and as predicted, we did not detect any Pax7 binding at enh-F (Fig 4B), supporting the hypothesis that Pax6 occupies this binding site in enh-F.

### Functional perturbation of upstream transcription factors disrupts enhancer activity

To further resolve key TF combinations required for *Chd7* enhancer activity, we used the chick CRISPR/Cas9 system [51] to knock down multiple TFs simultaneously. By co-electroporating enhancer reporter constructs with guide RNAs (gRNAs) and Cas9 plasmids, in a bilateral fashion [39] (S5B Fig), we could directly assess the effects of TF loss on enhancer activity (Fig 4C–4E).

As the predominant TF bound by all *Chd7* enhancers and a neural crest master regulator, we primarily considered knocking-out combinations of TFs that included *Sox10*. We also focused our functional assessments on the neural crest-specific enhancers, enh-A, enh-T, and enh-C. As described above, enh-T was bound by Sox10 and Tfap2B (Fig 4B), and in addition this enhancer was enriched for Sox2/3 binding motifs (Fig 4A). Similarity of binding motifs of closely related paralogues (for example, SoxB factors—Sox2/3, or SoxE factors—Sox8/9/10) can make it difficult to discern the specific factor occupying a particular site. However, given that enh-T is also active in the otic placode region, where *Sox3* is more highly expressed than *Sox2*, we hypothesised that SoxB binding site within the enh-T was occupied by Sox3. Using previously described gRNAs [32,37], we targeted *Sox10* in combination with *Sox3* and *Tfap2B* on the left side of the embryo and applied a control gRNA to the right side, with both sides also receiving Cas9-RFP and enh-T driving Citrine constructs. This perturbation resulted in a reduction of Citrine expression on the left side of the embryo, compared to the control right side, in the migrating neural crest cells, neural tube, and the otic region (Fig 4C and 4G).

Enh-C was the only *Chd7* enhancer with predominant activity in the trunk neural tube (Fig 2D). TF motif analysis revealed enh-C was enriched for Pax7 motifs (Fig 4A) and also contained motifs for Sox9 binding (S5A Fig). Both *Pax7* and *Sox9* are expressed in the trunk neural tube. Combined knock-out of *Pax7* and *Sox9* caused a reduction in enh-C activity specifically in the trunk neural tube at HH10 (Fig 4D and 4G).

We next sought to determine crucial TF combinations driving enh-A activity. Sox9 and Sox10 sites were both enriched in this enhancer (Fig 4A). Considering that SoxE factors are highly expressed in the neural crest, share similar binding motifs, and frequently collaborate we reasoned these could be core upstream factors for enh-A activity. We also identified Ets1 as a putative upstream factor (Fig 4A). Indeed, we found that simultaneous knock-out of *Sox9*, *Sox10*, and *Ets1* caused a profound reduction in enh-A activity in the migrating neural crest at 10ss (Fig 4E). SoxB sites were also enriched in enh-A; however, combined knock-out of these factors had no effect on enh-A driven Citrine expression, suggesting these factors act as canonical transcriptional repressors to inhibit enhancer activity in non-neural crest cells, consistent with their expression in neural tissue. In parallel, we generated a mutant construct of enh-A whereby all the Sox sites were mutated this resulted in reduced enhancer function (Fig 4F and 4G) reinforcing the notion that enh-A is primarily driven by Sox factors.

Using functional perturbation experiments corroborating our motif analysis and ChIP data, we demonstrated that activities of *Chd7* neural crest enhancers are indeed functionally dependent on neural crest transcription factors.

## Discussion

Transcriptional regulation of gene expression is mediated by *cis*-regulatory elements (enhancer and promoters) that integrate the action of upstream transcription factors upon the downstream target gene(s). Developmental genes are regulated by distally located enhancers containing cell type-specific transcription factor motifs facilitating spatial and temporal control of gene expression. Conversely, regulation of broadly expressed factors tends to be mediated by TSS proximal enhancers containing a different profile of ubiquitous motifs enabling stable core promoter interactions. Reports of tissue-specific regulation of omnipresent factors by distal acting enhancers are rare.

Despite its broad expression, heterozygous loss of *CHD7* in humans leads to CHARGE syndrome, a tissue-specific neurocristopathy affecting structures patterned by, or derived from, neural crest cells. Tissue-specific function of chromatin remodellers has previously been attributed to downstream interactions with tissue-specific transcription factors. For example, in Schwann cells, the transcription factor SOX10 recruits chromatin-remodeling complexes to *cis*-regulatory regions of *OCT6* and *KROX20* genes [52], whereas in Oligodendrocytes, OLIG2 is reported to direct SMARCA4/BRG1 to *cis*-regulatory regions of genes which control their differentiation [53]. Similarly, tissue-specific transcription factors were reported to directly recruit and target PRC1 to chromatin in megakaryocytic cells [54]. Furthermore, previous *CHD7* studies resolved downstream targets and partners for CHD7 activity, for example, CHD7 is reported to bind to enhancers of the neural crest transcription factor *FOXD3* in mouse neural crest stem cells along with OCT3/4, SOX2, and NANOG [30]. And in human neural crest cells CHD7 binds with PBAF at a distal regulatory element upstream of *SOX9* [26]. Here, we probed the intriguing possibility that neural crest enrichment of a chromatin remodeller such as *Chd7* may be controlled by tissue-specific *cis*-regulatory elements and associated upstream transcription factors. In doing so, we also established the position of *Chd7* in the neural crest gene regulatory network.

Consistent with previous work in the chick [55], we showed that *Chd7* expression initiates at HH8 along the cranial neural tube including pre-migratory neural crest cells. *Chd7* was up-regulated in neural crest cells throughout their emergence from the dorsal neural tube and during their subsequent migration. *Chd7* transcripts were also detected in placode derivatives including the eye and otic vesicles, in addition to the pharyngeal arches, dorsal root ganglia, and trigeminal ganglia. This pattern of *Chd7* expression is conserved in mice [27], zebrafish [56], and Xenopus [26].

Using regulatory data from isolated chick cranial and vagal neural crest cells, we identified a group of 11 novel *Chd7* enhancers whose in vivo collective activity fully recapitulated *Chd7* expression. While some enhancers display cranial neural crest specific activity, others were more broadly active in the head and/or trunk and vagal neural crest. Importantly, we found conserved chromatin accessibility profiles in our multiome data generated from early human embryonic cranial tissue. We also detected high levels of conservation in the underlying enhancer sequence which was reflected in conserved enhancer activity in vivo. Demonstrating the conservation of *Chd7* regulation during embryo development across amniotes and also supports the exquisite utility of the chick model for exploring human conditions. While many clinical CHARGE syndrome cases contain pathogenic variants in the *CHD7* ORF, it is highly plausible that many of the remaining unattributed cases would be caused by disruptions in *cis*-regulatory elements controlling *CHD7*. Thus, the enhancers identified here represent potential alternative screening sites for CHARGE syndrome individuals without pathogenic variants within the *CHD7* gene body.

Enhanceropathies are a group of clinical conditions caused by pathogenic variants or mis-regulation of enhancers. This can occur via direct mutation of the enhancer sequences, e.g., the point mutation in a sonic hedgehog enhancer that causes polydactyly [57], or mutation of upstream genes that produce proteins which regulate enhancers, e.g., Kabuki syndrome [58]. Interestingly, Kabuki syndrome is attributed to pathogenic variants of members of the polycomb repressive complex-2 (PRC2) [59] and has overlapping features with CHARGE syndrome [60].

It is therefore important to understand which motifs within an enhancer are required for transcription factor binding to induce enhancer activity. As such, we used transcription factor motif predictions to identify the core factors responsible for *Chd7* enhancer activity. In doing so, we reveal potentially sensitive sites for disease causing mutations. We validated motif predictions using ChIP assays and found the neural crest master regulator Sox10 binding at neural crest specific enhancers enh-A and enh-T. Functional relationships between *Sox10* and *Chd7* have been previously described. *Sox10* is dysregulated in *Chd7* morphant zebrafish [46] and co-IP experiments have shown Sox10 and Chd7 physically interact to regulate downstream targets [47]. Here, we extend this relationship by demonstrating that Sox10 also acts as an upstream driver for *Chd7* expression within the neural crest, mediated by at least 2 distal *cis*-regulatory elements, termed enh-A and enh-T. We also identified Tfap2B binding at enh-T. Tfap2B and Chd7 are known to interact in neural progenitor cells [61], but similarly to *Sox10*, *Tfap2B* is reported to be reduced upon loss of *Chd7* in that context. Our work demonstrates that Tfap2B is also driving *Chd7* enhancer activity in neural crest cells, potentially indicating the existence of positive feedback loops between Sox10/Tfap2B and Chd7. We also validated Pax7 binding enh-C, which is active throughout the trunk. This is the first report of Pax7 regulating *Chd7* and provides evidence of axial specific regulation of Chd7. Furthermore enh-C has a conserved and active counterpart in human, and pathogenic variants of PAX7 have recently been shown to affect craniofacial development [62], a phenotypic feature of CHARGE syndrome. Our data therefore offers an additional mechanism by which PAX7 functions to regulate normal neural crest biology.

Curiously, a significant proportion of *Chd7* enhancers contained retinoic acid receptor sites. RXRA has previously been described as a Chd7 interacting protein that is mutated in congenital heart disorders [40]. We also found a subset of Chd7 enhancers were enriched for Zic binding sites. Zic1/4 are responsible for cerebellar vermis anomalies in Dandy–Walker syndrome [63], which is also a phenotype of CHARGE syndrome. Zic1/2 gene-deficient mice share anterior hemisphere foliation defects with Chd7-deficient mice [64]. This demonstrates the overlap of phenotypes shared between neurocristopathies and other developmental defects and illustrates the need for more mechanistic understanding of the complex genetic relationships underlying such conditions, to improve diagnosis and clinical management.

In summary, here we describe upstream tissue-specific regulation of the chromatin remodeller, *Chd7*, during early chick neural crest development. We detail enhancer activity and cognate transcription factor binding, embedding Chd7 within the neural crest gene regulatory network, downstream of neural crest master regulators including Sox10, Tfap2B, and Pax7. Thus, providing a critical link between neural crest transcription factors and Chd7 activity, and ultimately providing putative mechanisms as to how they effectuate normal development of neural crest derived tissues. Furthermore, we demonstrate features of *Chd7* regulation are conserved in human, offering a potential resource for identifying pathogenic variants driving CHARGE syndrome as well as a novel mechanism for tissue specific activity of omnipresent factors.

## Methods

### Embryo collection

Chick embryos were harvested from fertilised Bovans Brown chicken eggs (Med Eggs) which were incubated at 37°C with approximately 60% humidity. Embryos were staged according to the Hamburger and Hamilton table of normal chick development [35]. All experiments were performed on chicken embryos younger than 12 days of development and as such were not regulated by the Animals (Scientific Procedures) Act 1986.

### Embryo preparation and electroporation

For ex ovo electroporation, gastrula stage (HH4) embryos were captured using the filter paper-based “easy-culture” [39,65]. Briefly, via a small hole made in the top hemisphere of the egg, the thick albumin was removed using blunt forceps, the thin albumin was collected in a 50 ml tube. The eggshell was removed until level with the yolk. Serrated forceps were then used to remove the residual albumin from the yolk, taking care not to damage the embryo. A piece of filter paper, approximately 1 cm square with a hole punched centrally was placed on the yolk with the embryo positioned in the middle. The filter paper was then cut along all 4 edges using dissecting scissors and the paper, with the embryo attached, was gently lifted off the yolk at a 45-degree angle. The paper was then inverted (embryo now ventral side up) and placed in a petri dish containing Ringer’s solution (125 mM NaCl, 1.5 mM CaCl_2_, 5 mM KCl, 0.8 mM Na_2_HPO_4_, 0.15 mM KH_2_PO_4_).

Plasmid(s) were combined and diluted to the desired concentration. pTK enhancer reporters were used at 2 μg/μl, ubiquitous electroporation control pCI-H2B-RFP (Addgene #92398) at 1.0 μg/μl, U6-3 gRNAs (Addgene #92359) at 0.5 μg/μl, and pCI-Cas9-RFP (Addgene #92397) at 1 μg/μl. Sufficient vegetable dye was added to visualise plasmids during electroporation. Plasmid(s) were injected into the cavity between the epiblast and underlying vitelline membrane to cover the entire epiblast and electroporated: 5 pulses of 5 V, 50 ms on, 100 ms off. Bilateral electroporation was used for perturbation experiments, whereby control and experimental regents are delivered to opposite sides of the primitive streak, providing ideal internal, stage matched controls for each experiment. Embryos were cultured on the reserved thin albumin at 37°C overnight to the desired stage [39]. Embryos were fixed in 4% paraformaldehyde (PFA) for 1 h at room temperature and subsequent washes in PBS prior to imaging.

For in ovo electroporation, fertilised eggs were incubated horizontally for approximately 40 h (to HH9). Using an 18 G needle and 5-ml syringe, 2 to 3 ml of albumin was removed from the egg via a small hole made at the pointed end of the egg, avoiding the yolk. A “window” was then cut at the top of the egg, approximately 1.5 cm × 0.8 cm in size. Having located the embryo in the egg, plasmids were injected into the entire length of the neural tube. Electrodes were placed laterally alongside the embryo and electroporated at 5 pulses of 10 V, 50 ms on, 100 ms off. A few drops of Ringer’s solution, supplemented with penicillin/streptomycin, was added on top of the embryo. The window was then sealed carefully with Sellotape, ensuring no gaps remained. Embryos were cultured at 37°C for 2 to 6 days. Embryos were extracted from the egg and fixed in 4% PFA for 1 h at room temperature and subsequent washes in PBS prior to imaging.

### Enhancer cloning and testing

Putative enhancer elements were cloned and tested individually as described below and in [38]. Putative enhancers were amplified from chick genomic DNA using primers containing specific “tails” (5′ TTTTTTCGTCTCgccagg n^20^, 3′ TTTTTTCGTCTCcaacag n^20^) to facilitate subsequent cloning into the pTK Citrine reporter vector using a modified GoldenGate [66] protocol. Gel-purified amplicons were combined with modified pTK Citrine reporter vector (Addgene #130513) with T4 DNA ligase and BsmBI restriction enzyme and subjected to a cycling reaction that allows simultaneous BsmBI digestion and T4-mediated ligation of the amplicon into the reporter vector (37°C 5 min, 16°C 10 min, ×15, 55°C 5 min, 80°C 5 min). The ligation/digestion reaction was then transformed into DH5a cells. Subsequent colonies were sequenced to ensure the expected amplicon was present. Endotoxin-free plasmid preparations (Qiagen endo-free maxi prep kit, Cat. #12362) were prepared for electroporation.

### Imaging analysis

Embryos were screened on an Olympus MVX10 stereomicroscope with 2.5× objective using Axio Vision 4.8 software. Zeiss 780 Upright confocal microscope was used for imaging at high cellular resolution. Embryos at HH8-12 were mounted on a microscope slide previously layered with electrical insulation tape (×2 layers), with rectangular gaps cut in with a scalpel, and covered with a cover slip, retaining sufficient fluid for optimal imaging. Older embryos were placed in a 35 mm glass bottomed imaging dish and secured with a few drops of low-melting point agarose.

### Hybridisation chain reaction

Fluorescent in situ hybridsation chain reaction was performed using the v3 protocol [34]. Briefly, embryos were fixed in 4% PFA for 1 h at room temperature. Older embryos (HH12-HH18) were bleached in 3% H_2_O_2_/8% KOH until any pigments were cleared. All embryos were dehydrated in a methanol series and stored at −20°C at least overnight. Following rehydration embryos were treated with Proteinase-K (20 mg/ml) for 2.5 to 25 min depending on stage (2.5 min for HH9-10, 25 min for HH12-HH15 embryos) at room temperature and postfixed with 4% PFA for 20 min at room temperature. Embryos were washed in PBST for 2× 5 min on ice, then 50% PBST/50% 5× SSCT (5× sodium chloride sodium citrate, 0.1% Tween-20) for 5 min on ice and 5× SSCT alone on ice for 5 min. Embryos were then pre-hybridised in hybridisation buffer for 5 min on ice, then for 30 min at 37°C in fresh hybridisation buffer. Probes were prepared at 4 pmol/ml (in hybridisation buffer), pre-hybridisation buffer was replaced with probe mixture and embryos were incubated overnight at 37°C with gentle nutation. Excess probes were removed with probe wash buffer for 4× 15 min at 37°C. Embryos were pre-amplified in amplification buffer for 5 min at room temperature. Hairpins were prepared by snap-cooling 30 pmol (10 μl of 3 mM stock hairpin) individually at 95°C for 90 s and cooled to room temperature for minimum 30 min, protected from light. Cooled hairpins were added to 500 μl amplification buffer. Pre-amplification buffer was removed from embryos and hairpin solution was added overnight at room temperature, protected from light. Excess hairpins were removed by washing in 5× SSCT 2× 5 min, 2× 30 min, and 1× 5 min at room temperature. Embryos were mounted on slides and imaged using Zeiss LSM 880 Upright confocal microscope. Images were processed using Zeiss Zen software, Z-stacks scans were collected at 6 μm intervals across approximately 70 to 200 μm, maximum intensity projections of embryo z-stacks are presented. Tile scanning was used and stitched using bidirectional stitching mode, with overlap of 10%.

### Biotin-ChIP-seq

To facilitate Biotin-ChIP, Avi-tagged transcription factor plasmids (Addgene #127775 Sox10, #127776 Tfap2B) were co-electroporated at 1.0 μg/μl with a pCI NLS-BirA-2A-mCherry plasmid (Addgene #127781) at 0.5 μg/μl into HH4 embryos (ex ovo) for collection at HH8 (Sox10) or HH8/9 (in ovo) for collection at HH18 (Tfap2B). The Biotin-ChIP-seq procedure is described below and here [48]. Approximately 15 embryos per experiment were harvested and their cranial (Sox10) or vagal (Tfap2B) regions (somites 1–7) dissected, providing approximately 100,000 cells of interest. Dissected embryonic tissues were dissociated in nuclei extraction buffer (NEB) (0.5% NP40, 0.25% Triton-X, 10 mM Tris-HCl (pH 7.5), 3 mM CaCl_2_, 0.25 M sucrose, 1 mM dithiothreitol, 0.2 mM phenylmethylsulfonyl fluoride, 1× proteinase inhibitor (PI)) in a glass dounce homogeniser. Cells were crosslinked in 1% formaldehyde at room temperature for 15 min and quenched with 125 mM of 1 M glycine for 5 min at room temperature. The crosslinker was washed out 3 times with 1× PBS/PI (1× PBS, 1× PI, 1 mM dithiothreitol, and 0.2 mM phenylmethylsulfonyl fluoride) and centrifuged at 2,000g for 4 min at 4°C. Pellets were snap-frozen and stored at −80°C until sufficient samples were collected. Pellets were thawed and resuspended in 1 ml NEB, centrifuged at 2,000g for 4 min at 4°C, and washed once with PBS/PI before nuclei lysis in SDS lysis buffer (0.7% SDS, 10 mM EDTA, 50 mM Tris-HCl (pH 7.5), 1× PI). Crosslinked chromatin was sonicated at 12A, 10× (10 seconds on, 30 seconds off) followed by 8A, 4× (30 seconds on, 30 seconds off) and run on a 1.5% agarose gel to ensure appropriate sheared DNA fragments. Sheared chromatin samples were pre-cleared in pre-blocked streptavidin beads (Dynabeads M-280 streptavidin beads, Invitrogen) overnight at 4°C on a rotator, and 1/20 of the chromatin was collected as an input fraction and stored at −80°C. Beads were washed with SDS wash buffer (2% SDS, 10 mM Tris-HCl (pH 7.5), 1 mM EDTA) at room temperature, followed by 4 radioimmunoprecipitation assay buffer (RIPA) washes (50 mM Hepes-KOH (pH 8.0), 500 mM LiCl, 1 mM EDTA, 1% NP40, 0.7% Na-deoxycholate, 1× PI) and one NaCl TE wash (1× TE (10 mM Tris, 1 mM EDTA), 50 mM NaCl) at 4°C. Samples were eluted from beads with SDS ChIP elution buffer (50 mM Tris-HCl (pH 7.5), 10 mM EDTA, 1% SDS) and crosslinking was reversed by overnight incubation at 65°C in the thermomixer at 1,000 rpm. Chromatin samples were then separated from streptavidin beads. Cellular RNA was digested with RNaseA (0.2 μg/ml) at 37°C for 1 h and cellular proteins were removed with proteinase K (0.4 mg/ml) at 55°C for 2 h. Samples and input DNA were then extracted using standard phenol:chloroform and ethanol precipitation protocol. Libraries were prepared using a MicroPlex Library Preparation v2 kit (Diagnode) with the number of cycles determined by the amount of starting material (Sox10 ChIP, 10 cycles, Tfap2B ChIP, 14 cycles) final libraries were quantified and sequenced using a NextSeq 500/550 High Output Kit v2 (75 cycles) on the NextSeq 500 sequencing platform.

### Genome editing

Guide RNAs were manually designed to target genes by searching for appropriate PAM (NGG) sites within the exon preceding the exon containing the DNA-binding domain of the protein. In this study, we used guide RNAs previously validated in our lab [32,37,51]. Guide RNAs were electroporated at 0.5 μg/μl with pCAG-Cas9-2A-Citrine at 1.0 μg/μl (Addgene #92358) [51].

### Cut and run

Cut and run was performed according to published protocols [49,50]. **Cell preparation:** Embryos (×10 per stage) were collected at the desired stage and placed in 1 ml NEB (0.5% NP40, 0.25% Triton-X, 10 mM Tris-HCl (pH 7.5), 3 mM CaCl_2_, 0.25 M sucrose, 1 mM DTT, 0.2 mM PMSF, 1× protease inhibitor tablet), following 10 strokes in a glass dounce homogeniser with pestle A on ice, the solution was centrifuged 600g for 3 min at room temperature in a low-binding 1.7 ml Eppendorf tube. Nuclei extraction buffer was removed and cells were washed twice with DIG-wash buffer (20 mM HEPES (pH 7.5), 150 mM NaCl, 0.5 mM Spermidine, 0.05% digitonin, 1× protease inhibitor tablet). **Binding cells to beads and antibody incubation:** Pelleted cells were then resuspended in 1 ml DIG-wash buffer and Concanavalin-A beads were added while shaking in an Eppendorf ThermoMixer at moderate speed. After 10 min rotating, the bead-cell solution was divided into aliquots, one for each antibody to be used. The beads were collected on a magnetic stand, DIG-wash buffer was removed and 150 μl of antibody buffer (2 mM EDTA in DIG-wash buffer) was added to each aliquot while shaking. Primary antibody was added at 1:110 dilution (αChicken Pax7 (DSHB), H3K27ac Abcam Ab4729) and samples were incubated overnight at 4°C with end-over-end rotation. **Binding Protein-A/G-MNase fusion protein:** Following a brief spin, beads were collected on a magnetic stand and washed twice with 1 ml DIG-wash buffer. While shaking, 150 μl of the Protein-A/G-MNase solution was added to the beads and incubated for 1 h at 4°C with rotating. Beads were collected on the magnetic stand and washed twice with DIG-wash buffer and resuspended in 100 μl DIG-wash buffer while shaking. Tubes were transferred to a cold block on ice, chilled to 4°C, 2 μl of 200 mM CaCl_2_ was added with shaking and promptly placed back in the cold block on ice for 30 min. While shaking 100 μl of STOP-buffer (340 mM NaCl, 20 mM EDTA, 4 mM EGTA, 0.05% Digitonin, 100 μg/ml RNase A, 50 μg/ml Glycogen) was added and incubated at 37°C for 30 min. **Collection of fragmented antibody bound DNA:** Beads were collected on a magnetic stand and the supernatant, now containing digested chromatin, was transferred to a new 1.7 ml low-binding Eppendorf tube. Chromatin proteins were degraded by adding 2 μl of 10% SDS, 2.5 μl of Proteinase-K (20 mg/ml), and incubated at 50°C for 1 h. DNA fragments were cleaned up using standard phenol:chloroform extraction and ethanol precipitation and resuspended in 20 μl water. Following quality control measures on Agilent Tapestation (high sensitivity) and Qubit, sequencing ready libraries were made using the NEBNext Ultra II kit and sequenced on the Illumina Next-seq 500/550 platform using High Output Kit v2 (75 cycles).

### Data analysis

#### 10× Single-cell RNA-Seq data analysis

Single-cell RNA-seq data was demultiplexed using 10X Genomics Cell Ranger v7.1.0 mkfastq [67] followed by alignment to the bGalGal1.mat.broiler.GRCg7b genome assembly with Ensembl 110 gene models and subsequent quantification using cellranger count function. A total of 5,669 cells were recovered, with 85,006 mean reads and 6,231 median genes per cell. Downstream analysis on the count matrix was completed using Seurat v5.0.0 [68] in R v4.3.1. The count matrix was filtered to remove barcodes containing fewer than 750 genes, greater than 10,000 genes, or greater than 2 percent mitochondrial gene counts. After filtering, the Seurat object was normalized using Seurat’s v2 SCTransform function, regressing out percent mitochondrial, and running through Seurat’s Principal Component Analysis for dimensionality reduction. Using the top 30 PCs, Seurat’s RunUMAP and FindNeighbors were run on the data before clustering the cells with resolution 0.4. Differentially expressed marker genes were calculated for these clusters via Seurat’s FindAllMarkers function and then used for the manual annotation of cluster identities. Raw and processed data is available from GEO GSE270155.

## Multiome data sets

Chd7 epigenomic locus was visualised using human embryonic multiome data sets generated for a different study by D. Fountain, University of Oxford. Samples were obtained from donors in accordance with ethically approved study REC 96/085 (University of Cambridge and Cambridge University Hospitals NHS Foundation Trust). Samples were transferred under a Material Transfer Agreement and stored and processed in accordance with ethically approved study REC 22/PR/0630 (University of Oxford) on HTA-licensed premises (license number 12433). Nuclei from 4 cranial samples across 3 Carnegie Stages (CS16, CS18, CS18, CS19) were analysed using the Chromium 10X Next GEM Single Cell Multiome ATAC + Gene Expression kit. Libraries were sequenced in accordance with 10X recommendations and processed with Cellranger v7.1.0. One library was rejected due to poor median genes per nuclei. Three libraries were included totalling 14,290 nuclei with a final median genes of 2,770 per nuclei and median depth of 129,399 reads per nuclei for gene expression libraries. Pooled libraries were demultiplexed into individual samples using genetic demultiplexing. To facilitate this, bulk RNA-seq libraries were generated and sequenced to a targeted depth of 50 million reads. Variant calling and genotyping were performed in accordance with the GATK RNAseq short variant discovery (SNPs + Indels) pipeline. Multiome samples were demultiplexed using Souporcell within Demuxafy v2.0.1. Ambient RNA was detected and removed using Cellbender v0.3.0. Doublets were detected based on genotype using Souporcell and separately using scDblFinder both using Demuxafy v2.0.1. Libraries were merged resulting in 11,719 nuclei and clustered with the standard Seurat v4.9.9.9058 pipeline (resolution 1.0). Clusters were annotated manually using the FindAllMarkers function (logfc threshold 0.25, min pct 0.25). This data can be accessed here GSE262042.

## ChIP-seq and Cut and Run analyses

Avi-tagged Sox10 biotin ChIP sequencing and Cut and Run samples were demultiplexed and the resulting files were merged. Previously published biotin-ChIP-seq data for avian TFAP2b from HH18 embryos [37] were downloaded from the GEO database (GSE125711). We evaluated read quality using FastQC v0.11.4 [69]. Adaptor sequences were trimmed using TrimGalore v0.4.1 and then mapped to the chicken genome galGal5 assembly using bowtie2 v2.3.5 [70]. Sequence alignment/map files were compressed to the binary version (BAM) for downstream analysis. Only aligned pairs were retained based on binary alignment map (BAM) flags, and mitochondrial reads were removed from the BAM file using Samtools (v1.3) [71]. PCR duplicates were then removed using PicardTools (v1.83) and only uniquely mapped reads were retained. A custom Perl script was used to generate smoothened genome browser tracks in BigWig format for data visualisation on the UCSC Genome Browser. The Pax7 Cut&Run and Sox10 biotin-ChIP data can be accessed here GSE261486.

Similarly, we downloaded raw fastq files of human ChIP-seq for histone posttranslational modifications previously published [44] from the GEO database (GSE70751). These files were processed as described above, with the exception that reads were mapped to the human genome assembly, hg38. Peaks were called for each ChIP-seq sample using MACS2 (v.2.0.10) [72]. Differential accessibility analysis was carried out in R (v.3.2.1) using the DiffBind package (v1.10.2). Differential accessibility across samples was calculated using a negative binomial distribution model implemented in DEseq2 (v1.4.5). The threshold FDR < 0.1 and Fold enrichment >1 was used to define differentially bound peaks over the input control.

## Motif enrichment within selected enhancers

To identify motifs enriched within *Chd7* enhancers, we used a list of 49 position weight matrixes (PWMs) from the HOCOMOCO v.11 database [73] previously filtered on their relevance to neural crest transcriptional regulation based on their enrichment in differentially accessible chromatin and expression of their cognate TFs in avian neural crest single-cell transcriptomes (described in detail in [32]). Homer (v.4.7) annotatePeaks.pl was used to screen such motifs within avian and human *Chd7* enhancers. We used the highest log-odds score within a peak as evidence of motif enrichment within selected *Chd7* enhancers.

## Supporting information

S1 File Genomic coordinates of all tested enhancers. (XLSX)

S2 File Phastcons scores of enhancer conservation. (XLSX)

S1 Fig Gene expression in single-cell Multiome RNA-seq data. (A) Violin plots of other chromatin remodellers and transcription factors (B) expressed across scRNA-seq clusters. (TIF)

S2 Fig In vivo activity of *Chd7* enhancers. Chick *Chd7* enhancers, as indicated by top boxes shown at alternative stages. Additional enhancers; B1, B2, I, J, O, Q are also shown. (TIF)

S3 Fig Co-localisation of enhancer activity with neural crest marker genes. (A, B) enh-A shown with HCR for *Sox10* at HH12 and HH15, respectively. Ai, Bi, and Bii show transverse sections through A and B as indicated by the white dashed lines. (C, D) enh-T with *Sox10* expression at HH12 and HH18, respectively. Ci and Di show sections as indicated by white dashed line. (E, F, G) enh-C, enh-D, and enh-H, respectively, all at HH12 with *Pax7 or Sox10* expression as indicated. (Ei, Fi, Gi) transverse sections of E, F, and G as indicated by white dashed lines.
(TIF)

S4 Fig Epigenomic profiles of human CHD7 locus. (A) Top left; UMAP plots depicting 33 clusters resolved from 3 samples totalling 14,290 nuclei top right; sample origin of cells, bottom left; feature plot of Chd7 expression across all clusters. (B) Feature plots of CHD7, other chromatin remodellers and transcription factors expressed across the scRNA-seq data from human Multiome data. (C) UCSC genome browser view of the CHD7 locus showing ATAC-seq and histone ChIP-seq data from human in vitro derived cranial neural crest cells [44].
(TIF)

S5 Fig Transcription factor motif enrichment in CHD7 enhancers. **(A)** Heatmap of predicted transcription factor motifs identified in *Chd7* enhancers using HOMER. (B) Schematics depicting biotin-ChIP assay (top panel) and bilateral electroporation (bottom panel). (C) In vivo activity of Sox10-enh99 used to drive Avi-tagged Sox10 for biotin-ChIP.
(TIF)

## Abbreviations

DRG: dorsal root ganglia
gRNA: guide RNA
GWAS: genome-wide association studies
HCR: hybridisation chain reaction
NEB: nuclei extraction buffer
PFA: araformaldehyde
PI: proteinase inhibitor
PWM: position weight matrix
RBC: red blood cell
TAD: topologically associating domain
TF: transcription factor
WGCNA: weighted gene co-expression network analysis
