# Supplementary figures and images for: Chromatin remodeller Chd7 is developmentally regulated in the neural crest by tissue-specific transcription factors
Source: PLoS Biol. 2024 Oct 17;22(10):e3002786. doi: 10.1371/journal.pbio.3002786 (PMC11521297; doi:10.1371/journal.pbio.3002786)

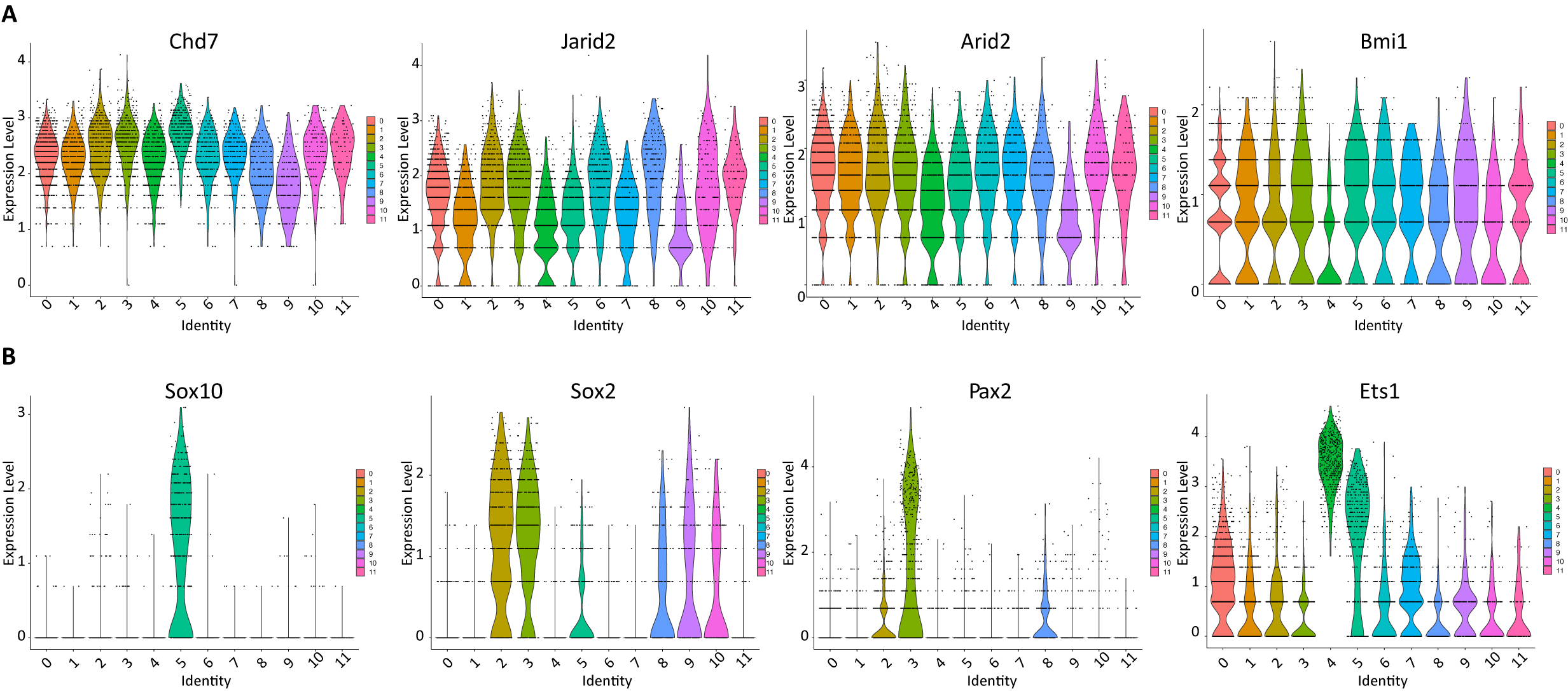

Supplement: S1 Fig — (TIF) [file pbio.3002786.s003.tif]

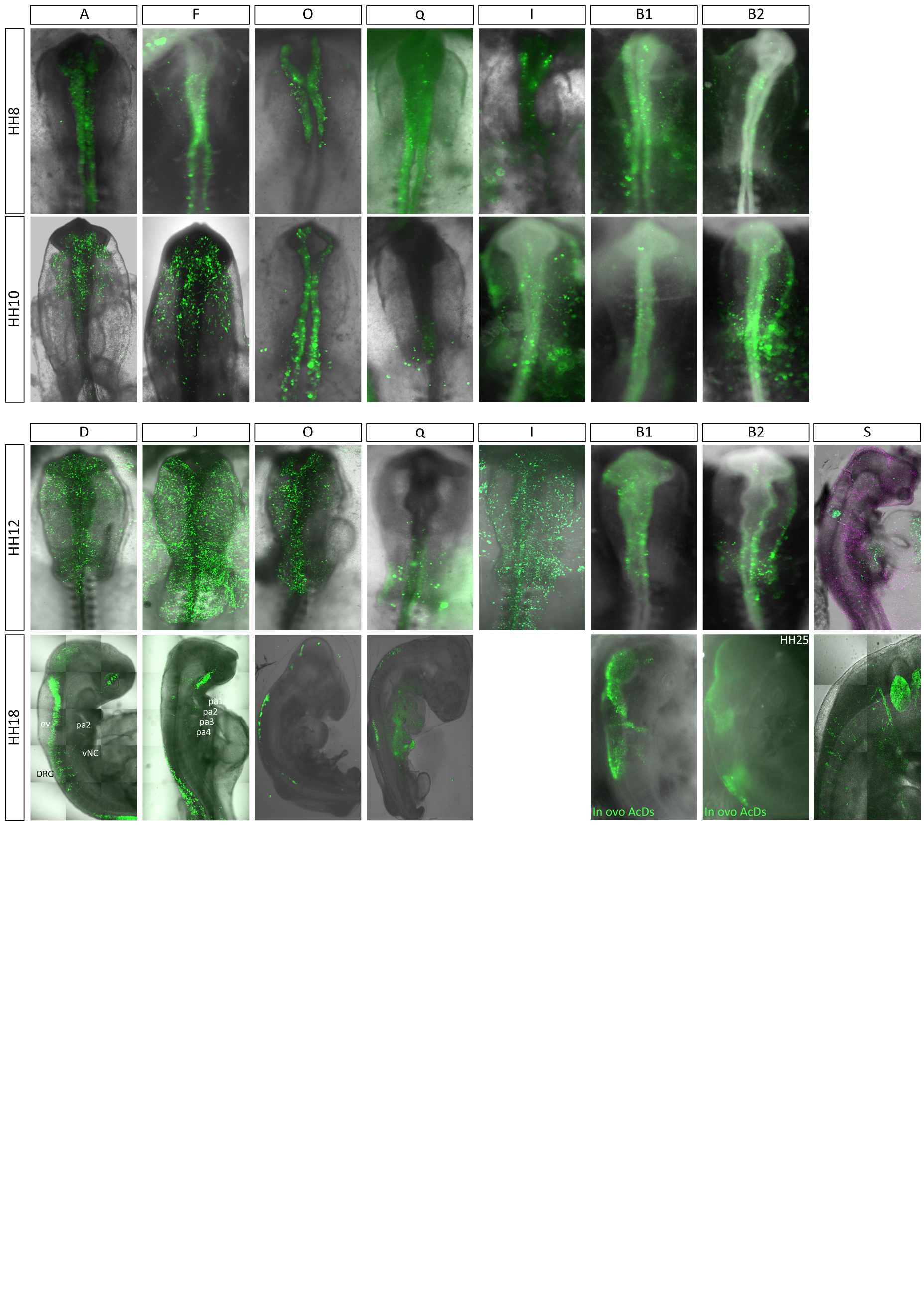

Supplement: S2 Fig — (TIF) [file pbio.3002786.s004.tif]

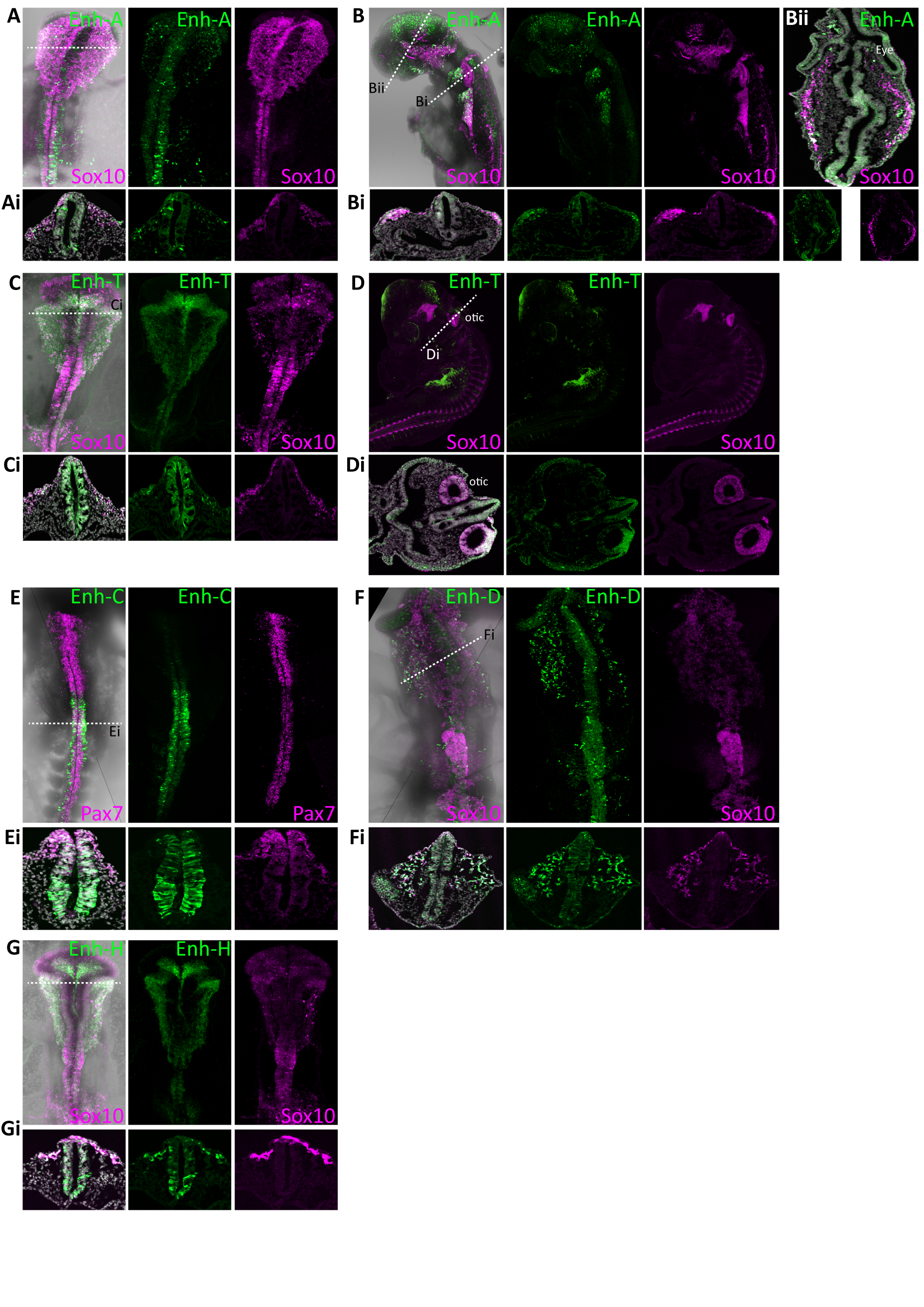

Supplement: S3 Fig — (A, B) enh-A shown with HCR for Sox10 at HH12 and HH15, respectively. Ai, Bi, and Bii show transverse sections through A and B as indicated by the white dashed lines. (C, D) enh-T with Sox10 expression at HH12 and HH18, respectively. Ci and Di show sections as indicated by white dashed line. (E, F, G) enh-C, enh-D, and enh-H, respectively, all at HH12 with Pax7 or Sox10 expression as indicated. (Ei, Fi, Gi) transverse sections of E, F, and G as indicated by white dashed lines. (TIF) [file pbio.3002786.s005.tif]

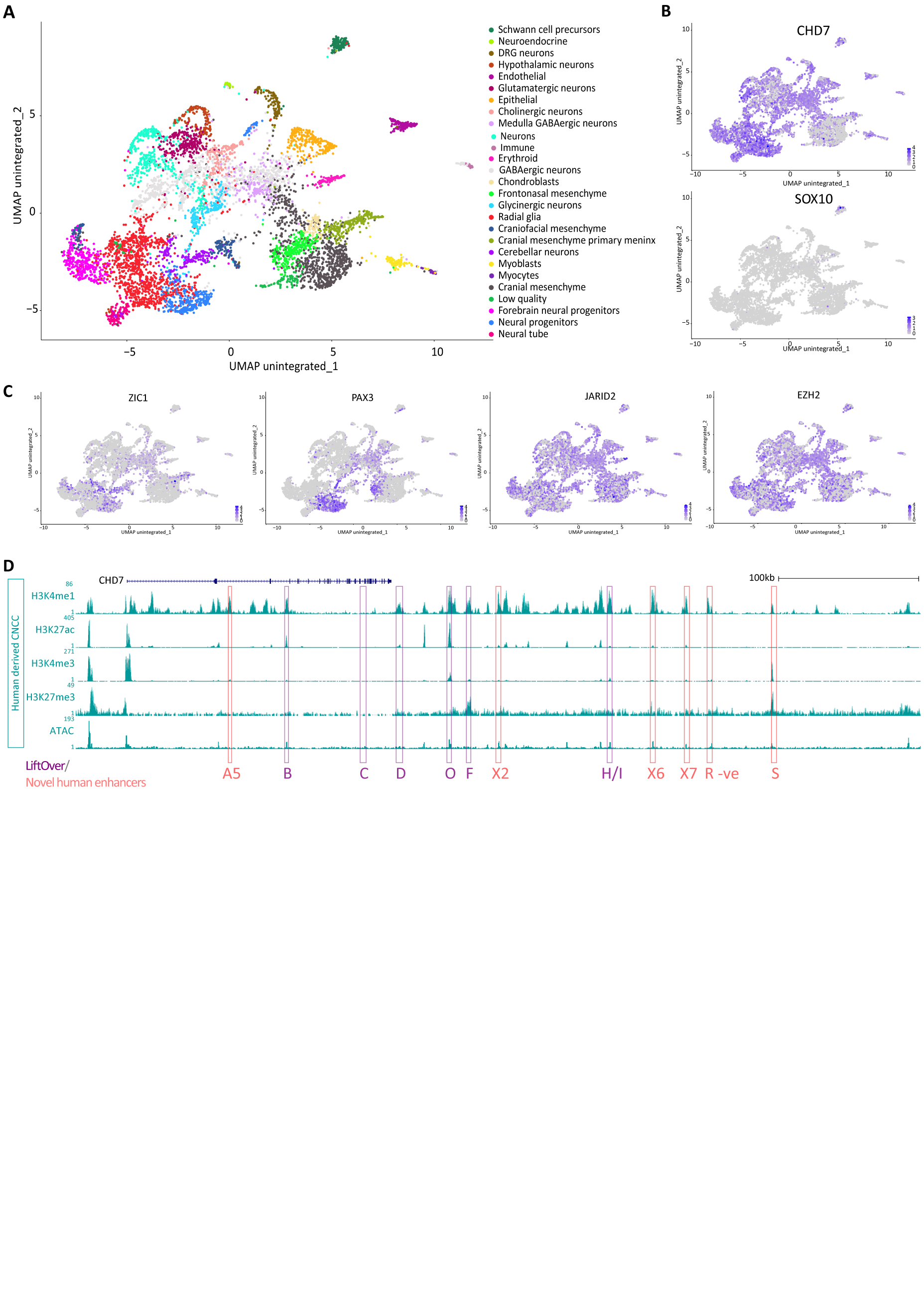

Supplement: S4 Fig — (A) Top left; UMAP plots depicting 33 clusters resolved from 3 samples totalling 14,290 nuclei top right; sample origin of cells, bottom left; feature plot of Chd7 expression across all clusters. (B) Feature plots of CHD7, other chromatin remodellers and transcription factors expressed across the scRNA-seq data from human Multiome data. (C) UCSC genome browser view of the CHD7 locus showing ATAC-seq and histone ChIP-seq data from human in vitro derived cranial neural crest cells [44]. (TIF) [file pbio.3002786.s006.tif]

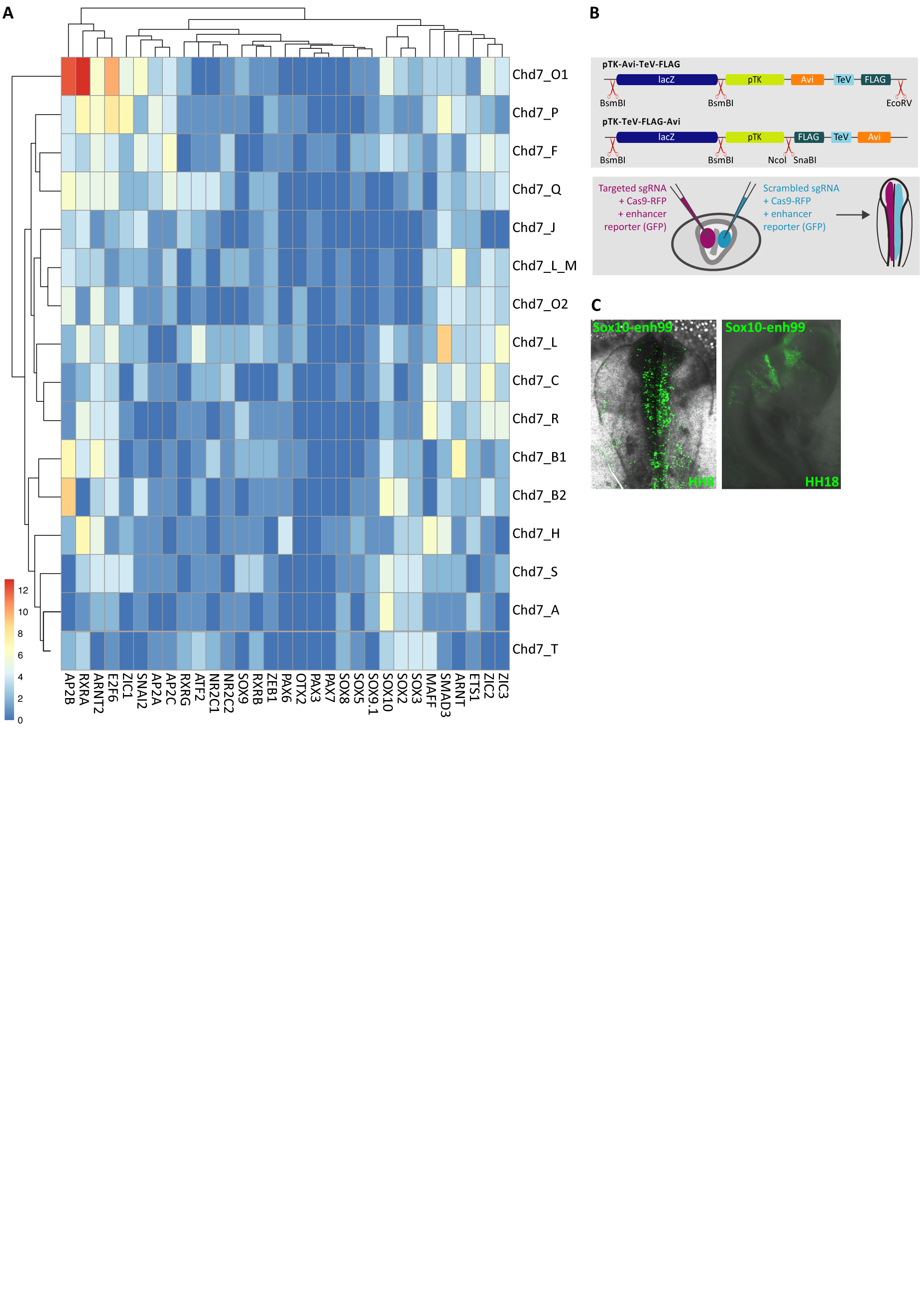

Supplement: S5 Fig — (A) Heatmap of predicted transcription factor motifs identified in Chd7 enhancers using HOMER. (B) Schematics depicting biotin-ChIP assay (top panel) and bilateral electroporation (bottom panel). (C) In vivo activity of Sox10-enh99 used to drive Avi-tagged Sox10 for biotin-ChIP. (TIF) [file pbio.3002786.s007.tif]
